# Impact of different strategies to control *Plasmodium *infection and anaemia on the island of Bioko (Equatorial Guinea)

**DOI:** 10.1186/1475-2875-5-10

**Published:** 2006-02-06

**Authors:** Gema Pardo, Miguel Angel Descalzo, Laura Molina, Estefanía Custodio, Magdalena Lwanga, Catalina Mangue, Jaquelina Obono, Araceli Nchama, Jesús Roche, Agustín Benito, Jorge Cano

**Affiliations:** 1 Centro Nacional de Medicina Tropical. Instituto de Salud Carlos III.c/Sinesio Delgado, 6, P.O. Box 28029, Madrid, Spain; 2 Centro de Referencia para el Control de Endemias. Centro Nacional de Medicina Tropical, Instituto de Salud Carlos III, Bata, Equatorial Guinea

## Abstract

**Background:**

On the island of Bioko (Equatorial Guinea), insecticide-treated nets (ITNs) have been the main tool used to control malaria over the last 13 years. In 2004, started an indoor residual spraying (IRS) campaign to control malaria. The purpose of this study is to asses the impact of the two control strategies on the island of Bioko (Equatorial Guinea), with regards to Plasmodium infection and anaemia in the children under five years of age.

**Methods:**

Two transversal studies, the first one prior to the start of the IRS campaign and the second one year later. Sampling was carried out by stratified clusters. Malaria infection was measured by means of thick and thin film, and the packed cell volume (PCV) percentage. Data related to ITN use and information regarding IRS were collected. The Pearson's chi-square and logistic regression statistical tests were used to calculate odds ratios (OR)

**Results:**

In the first survey, 168 children were sampled and 433 children in the second one. The prevalence of infection was 40% in 2004, and significantly lower at 21.7% in 2005. PCV was 41% and 39%, respectively. 58% of the children surveyed in 2004 and 44.3% in 2005 had slept under an ITN. 78% of the dwellings studied in 2005 had been sprayed. In the 2005 survey, sleeping without a mosquito net meant a risk of infection 3 times greater than sleeping protected with a net hanged correctly and with no holes (p < 0.05).

**Conclusion:**

IRS and ITNs have proven to be effective control strategies on the island of Bioko. The choice of one or other strategy is, above all, a question of operational feasibility and availability of local resources.

## Background

The fight against malaria in Sub-Saharan Africa has to be transectoral and tackled from the clinical, social and vectorial points of view, involving the local health structure (primary health care systems, national anti-malaria and vector control programmes) and international support programmes [1-3].

The vector control campaigns are based on environmental sanitation and suitable environmental management, the implementation of educational programmes and the use of insecticides, either to impregnate fabrics (mosquito nets, curtains....) or to spray (indoors and outdoors). The majority of these directives are currently the basic pillars of the fight against vectors, except for the use of pesticides, which has to be highly regulated and their handling subject to strict control, according to the recommendations of the WHO [4].

Despite the good results from controlling the transmission during the 50 s and 60 s with widespread spraying activities with insecticides from the organochlorine family (such as DDT) in various regions of the world [5], this strategy has not proven to be as effective in highly endemic African countries and has led to the emergence of factors that limit the success of the vector control campaigns: (i) appearance of vector populations that are physiologically resistant to organochlorine insecticides, and, in certain countries, resistance to pyrethroid and carbamate insecticides [1,6,7], (ii) social rejection of fumigation campaigns, mainly inside the home, and to toxic side effects from their use [8], (iii) changes in the feeding and resting behaviour of the vector populations exposed to surfaces treated by excito-repellent insecticides (DDT, pyrethroids) [8,9] and (iv) damage to species that act as natural predators of the vectors [10].

Insecticide-treated nets (ITNs) appeared to be an alternative to indoor residual spraying (IRS) and proved to be a good tool to reduce the morbidity-mortality among the most vulnerable groups (children and pregnant women) [11-15]. The first trials resulted in widespread control campaigns based on the use of ITNs [16-18]. In general, these campaigns led to the conclusion that ITNs are the ideal tool to control malaria transmission, in particular in countries with a high endemicity, provided that they are used correctly and that a high population coverage is reached [19].

Despite what has already been stated, control campaigns based on IRS are being implemented in many countries of Sub-Saharan Africa. These are obtaining excellent results, in particular in those countries where the transmission of malaria is not constant or seasonal [20,21].

On the island of Bioko (Equatorial Guinea), ITNs have been the main tool used to control malaria over the last 13 years (1991–2004). At the start of 2004, a private initiative, funded by oil companies, was set up under the aegis of Equatorial Guinea's Ministry of Health, to implement a huge campaign to control malaria on the island. The campaign was based on IRS with pyrethroids.

The aim of this study was to assess the impact of the two control strategies currently used on the island of Bioko with regards to Plasmodium infection and anaemia in children under five years of age.

## Methods

### Study area

**Figure 1:**
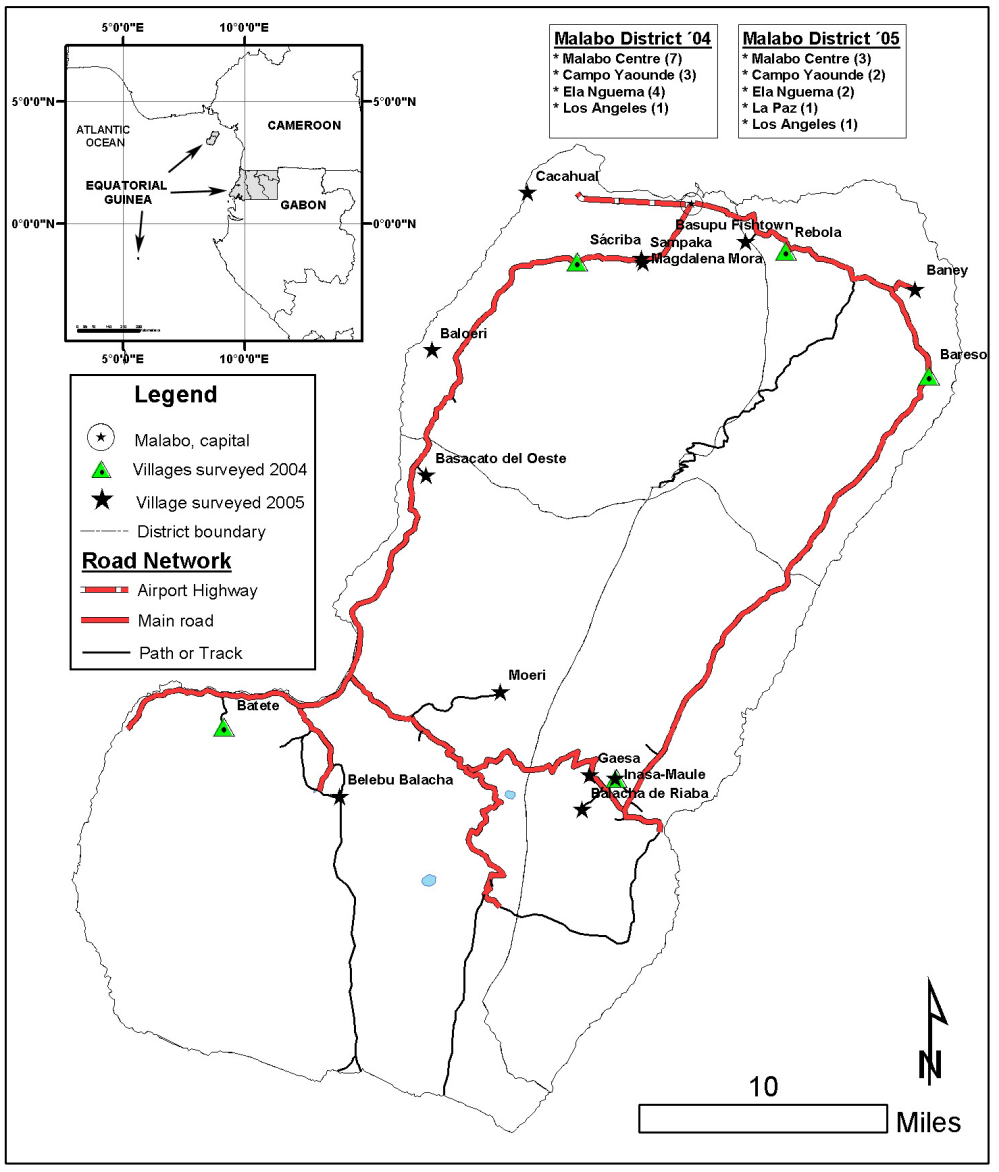
Map of the island of Bioko and distribution of conglomerates (districts and villages) in 2004 and 2005.

Equatorial Guinea, a Central African country located in the Golf of Guinea, consists of an island region and a continental region. Bioko (N 3° 43' and E 8° 43') is the main island, measuring 2017 km2 and with approximately 60,000 inhabitants. It is 30 km from the coast of Cameroon and its landscape is very mountainous, due to the volcanic origin of the island, with two main peaks: Mount Basilé (3012 m) in the north and Mount Biao (2009 m) in the south (Figure 1). There are two climatic seasons on the island of Bioko: a dry season, which lasts from December to May, and a rainy season from June to November. Bioko's annual rainfall reaches 2,120 mm in 123 days. The relative humidity ranges between 70% and 100% throughout the year. The average temperature is 25°C, with the minimum ranging between 17°C and 21°C and the maximum between 29°C and 30°C, depending on the location and the season [22].

Malaria in Equatorial Guinea is hyper to holoendemic, which is also the case in neighbouring countries such as Cameroon [23], with 55% of children under five years of age infected and with a splenic index over 50% in children between two and nine years of age [24]. On the other hand, studies recently conducted in Bioko have shown that the entomological inoculation rate (TIE) for the two vector species on the island, *Anopheles gambiae s.s*. and *Anopheles funestus*, was between 242.7 and 281 infective bites per year in the case of the first species, and between 317 and 787.6 for the second [25].

### Sampling method

Two transversal studies were conducted: the first one prior to the start of the IRS (February 2004) and the second, one year later (February 2005). Sampling was carried out by stratified cluster survey: multi-stage in 2004 and two-stage in 2005. During the first survey, one child per household was chosen at random (non self-weighted sample), while all the children from the selected households in the second survey were sampled (self-weighted sampling by cluster).

### Data collection and laboratory analysis

A blood sample was taken from the selected children to determine the malaria infection by means of the microscopical examination of a stained thick and thin film. Each sample was studied by two qualified laboratory technicians and a third technician was called in when there was a discrepancy in the result. Temperature and packed cell volume (PCV) percentage were also measured, with anaemia defined as a PCV percentage below 30% [26]. A curative dose of sulphadoxine-pyrimethamine was given to all the children taking part in the study. On the other hand, a short questionnaire was used to gather data relating to the use of ITNs and the characteristics of the dwellings. The second survey also collected information regarding the IRS

### Statistical analysis

A database was created in Access 2000 to computerize the data collected on the form. STATA 8.2 statistical software was used to analyse the data. The non self-weighted design of the sample was taken into account and the data was weighted in accordance with the selection probabilities. The cluster design effect was also taken into account when calculating the 95%confidence intervals. The comparisons between groups were carried out using the Pearson's chi-square test. Logistic regression analysis was used to calculate the Odds Ratios (OR) of the infection's potential predictors and to compare the results between the two surveys. A p-value <0.05 was considered to be statistically significant.

## Results

During the study conducted in February 2004, the number of selected households was 168: 143 in the urban area and 25 in the rural area, and 168 children under 5 were surveyed. In February 2005, the number of households selected for the study came to 279: 150 in the urban area and 129 in the rural area, and 433 children were surveyed.

Out of the 162 blood samples taken in 2004 (six refused to have their blood taken), 66 were positive for malaria, meaning a prevalence of 40% [95% IC: 26–53%]. In 2005, the prevalence was significantly lower at 21.7% [95% IC: 13.1–30.3%], based on the 432 samples collected (114 positive). When the crude parasite rate (CPR) for the two years is compared, there are significant differences depending on location, age and use of nets. However, there were no significant differences in the percentage of children with anaemia (Table 1). The majority of infections was monospecific for *Plasmodium falciparum*, 95% in the first study and 86% in the second.

**Table 1:** CPR and PCV for 2004 and 2005 in relation to the different study variables

		**CPR**			**Anaemia (PCV <= 30)**		
		**2004**	**2005**	**p**	**2004**	**2005**	**p**

**Global**		40% (26–53%)	22% (13–30%)	**<0.05**	41% (34–48%)	39%(23–54%)	>0.05

**Location**	**Urban**	39%(25–54%)	21% (17–24%)	**<0.05**	39%(32–47%)	35%(13–56%)	>0.05
	**Rural**	44%(21–66%)	23% (2–44%)		57%(34–79%)	45%(21–68%)	

**Age**	**< 1 year**	21%(2–41%)	8% (2–14%)	**<0.05**	58%(44–71%)	37%(19–54%)	>0.05
	**1 to 3 years**	43%(28–57%)	18% (8–28%)		34%(21–47%)	40%(25–54%)	
	**3 to 5 years**	55%(34–76%)	36% (17–55%)		32%(15–48%)	39%(14–64%)	

**Mosquito net**	**YES**	34%(16–52%)	11% (3–19%)	**<0.05**	45%(33–57%)	31%(10–51%)	>0.05
	**NO**	49%(33–65%)	30% (21–40%)		35%(27–44%)	44%(30–57%)	

**Spraying**	**YES**	-	20% (10–29%)	-	-	36%(20–52%)	-
	**NO**	-	29% (15–44%)	-	-	45%(20–71%)	-

The adults and legal guardians were asked about the use of mosquito bed nets by the children in question. 58% (95% IC: 48–68%) of the children surveyed in 2004 and 44.3% (95% IC: 31–57.7%) of those surveyed in 2005, had slept under a mosquito bed net the previous night. There were significant differences in the use of the mosquito net between the older and younger children, but no differences were found according to location in either of the two surveys (Table 2).

**Table 2:** Mosquito net users by age group and location

		**2004**		**2005**	
		***Frequency***	***Percentage***	***Frequency***	***Percentage***

Age*	**Under 1 year**	33	82%(67–98%)	44	49% (35–62%)
	**1 to 3 years**	23	47%(30–63%)	72	49% (33–65%)
	**3 to 5 years**	24	55%(37–73%)	48	35% (19–51%)

**Location**	**urban**	78	60%(49–71%)	109	48% (33–62%)
	**rural**	9	41%(24–59%)	55	40% (13–67%)

*p < 0.05

In contrast to to the first survey, a team member checked the quality of the usage of mosquito nets during the 2005 survey: whether the nets were correctly hung or not, if them were in the correct position and whether or not they had any holes. Only 4% (12) of the mosquito nets were not hung. With respect to hanging, 13 were incorrectly hung or were too small compared to the size of the bed (5%). 25% of the mosquito nets checked had holes.

On the other hand, 78% of the dwellings studied in 2005 had been fumigated with insecticide the previous year and 31% (52) of those had been fumigated more than once. In order to determine the impact of using mosquito nets on the CPR among the children surveyed in 2005, three groups were established: children not protected by a mosquito net (268), children protected by a net without holes and correctly hung (107) and children protected by nets that were not hung correctly and/or had holes (57). There was significant difference of the CPR of these three groups. When the CPR of the surveyed children was compared to the IRS, it was noted that there were no significant differences in the CPR of the children from fumigated homes with respect to the CPR of the children from non-fumigated homes, 20% and 29%, respectively (Table 3). Neither were there are significant differences between the dwellings that had been sprayed various times (13%), with those that had only been fumigated once (24%).

**Table 3:** CPR according to use of mosquito net and spraying (2005)

		***Percentage***	***L. I. (IC 95%)***	***L. S. (IC 95%)***
Mosquito net*	**Well hung and without holes**	11%	3%	20%
	**Do not use net**	30%	21%	40%
	**Badly hung and with holes**	12%	0%	24%

**Spraying**	**Dwelling sprayed**	20%	10%	29%
	**Dwelling not sprayed**	29%	15%	44%

*p < 0.05

The multivariant OR were calculated for the predictor factors of the infection by *Plasmodium *(Table 4). Children between three and five years of age showed 5 (2004) and 8.7 (2005) times more risk of being infected than those under one year of age (p < 0.05). The risk for children between one and three was 2.6 (2004) and 3 (2005) times greater (not significant). On the other hand, in the 2005 survey, sleeping without a mosquito net meant a risk of infection three times greater than sleeping protected with a net that was hung correctly and with no holes (p < 0.05). Sleeping under a badly hung or broken net also meant a higher risk, although this was not significant.

**Table 4:** Potential factors for suffering from malaria

		**2004**			**2005**		
**Predictors**		**n**	**OR**	**95% IC**	**n**	**OR**	**95% IC**

Place of residence	urban	138	1	-	232	1	-
	rural	24	1.2	0.2–7.3	200	1.3	0.5–3.2

Sex	man	82	1	-	225	1	-
	woman	80	0.9	0.3–2.1	207	0.6	0.3–1.2

Age	Under 1 year	41	1	-	95	1	-
	1 to 3 years	60	2.6	1–6.7	178	3.0	0.8–12.0
	3 to 5 years*	**47**	**4.9**	**1.2–19.7**	**159**	**8.7**	**3.7–20.8**

Sleeps under net (2004)	Yes	83	1	-	-	-	-
	No	76	1.2	0.5–3.2	-	-	-

Sleeps under net (2005)	Hung correctly without holes	-	-	-	107	1	-
	No*	**-**	**-**	**-**	**267**	**3.3**	**1.6–6.6**
	Incorrectly hung or with holes	-	-	-	57	1.5	0.6–4.2

Indoor fumigations	no	-	-	-	100	1	-
	yes	-	-	-	325	0.7	0.3–1.8

Number of fumigations	1	-	-	-	343	1	-
	More than 1	-	-	-	88	0.4	0.1–2.4

Type of building	Blocks or bricks	78	1	-	233	1	-
	wood	75	1.5	0.5–3.9	170	1.8	0.9–3.4
	others	9	4.1	0.5–34	29	0.7	0.2–2.4

*p < 0.05

## Discussion

The transmission of malaria in Equatorial Guinea is stable. There may be some seasonal variations in the transmission model, but there were no significant variations from one year to another [24]. Therefore, the fact that there was significant difference in the infection prevalence from one year to the next may be attributed to the introduction of a new variable that had a direct impact on the transmission. When the percentage of ITNs users among children was analysed, there was no significant difference between 2004 and 2005, although the percentage of users was slightly higher in 2004. This data should be interpreted with caution, as there was no control during the first survey, to check whether or not a child was really protected by a mosquito net while he slept or to establish the condition of the net. During the second survey, it was noted that a small percentage of the ITNs were not hanging at all or were not hung correctly, which suggests that the percentage of children protected by a mosquito net in the 2004 survey was overestimated. It is logical to believe that the dramatic reduction noted in the CPR over these two years is due to the implementation of the widespread indoor spraying campaign that took place during 2004, subsequent to the first survey. During a historical review of the malaria control activities based on IRS in countries in southern Africa [20], a dramatic reduction was noted in the case incidence, in particular shortly afterwards these campaigns had been implemented. In all the cases, even in unstable and low endemic malaria conditions, the transmission was never completely eradicated [27,28]. Similar results were noted in pilot projects started between 1950 and 1960 in tropical countries, such as Cameroon, Rwanda, Burundi, Tanzania, Kenya and Uganda [29,30]. Apart from the malaria indexes being reduced, there was always a dramatic reduction in the vector populations.

A factor to be taken into account with respect to the success of the control campaigns based on IRS would be the resting habit of the vector species [1,31]. On the island of Bioko, *An. gambiae s.l*. and *An. funestus *are the species responsible for the transmission. During a longitudinal study conducted on the island between 1998 and 1999, it was noted that both species showed markedly endophilic behaviour (resting indoors) during the dry season and exophilic behaviour (resting outdoors) during the rainy season [25]. As the dwellings were sprayed for the first time at the end of the dry and the start of the rainy season, it was guaranteed that the vector populations would be in contact with the recently treated surfaces.

On the other hand, a clear association between sleeping or not under a mosquito bed net and Plasmodium infection was noted in both surveys. This data coincides with the results obtained in all the experimental studies conducted with ITNs [11,12,14,15,18]. The fact that no major differences were found in the CPR between those children who slept under a mosquito net without holes and hung correctly and those that slept under a mosquito net with holes and/or badly hung, could be due to their being treated with insecticide. The majority of the tests conducted with mosquito bed nets have shown that the degree of protection of the treated mosquito nets is greater than that of the non-treated, even when they have holes [32]. In the present study, data relating to the re-impregnation or purchase date of the mosquito net was not gathered, which would have been information that could have corroborated the above hypothesis.

The difference found in the risk of infection between the different age groups could be associated to differences in the immunological status and the use of ITNs. It is known that, during the first months of life, the risk of infection is lower because there is still a degree of immunity from the mother. The risk of infection first increases with age and then decreases when the individual himself reaches a degree of immunity due to the numerous contacts with the parasite [33]. With respect to the use of mosquito nets, it was noted in the two surveys that the proportion of mosquito net users among children under one year of age is higher than the proportion of users in the group between three to years of age, which could also explain the difference in the CPR between those two age groups.

With respect to the prevalence of anaemia, a significant reduction was not noted as the result of using ITNs or IRS, contrary to what was seen in other studies [14,15]. The type of study carried out was probably not suitable to determine the impact of these two strategies on the blood haemoglobin levels. In areas of intense transmission, the majority of the cases of severe anaemia due to malaria would be associated with episodes of malaria, due to re-infections or to a poor reaction to the treatment [34,36]. Therefore, a longitudinal study would be more appropriate to evaluate the impact of these two strategies with respect to haemoglobin levels [15,35,36].

## Conclusion

IRS and ITNs on the island of Bioko have proven to be effective control strategies. In some trials carried out with ITNs, where high population coverage was reached, a similar community protection effect was noted to that obtained using IRS [14,15,37]. The health impact of the two strategies is fully proven. The choice of one or other strategy is, above all, a question of operational feasibility and availability of local resources [5]. A further two fundamental aspects when selecting one or the other would be: the degree of social acceptance or "prestige" of both strategies, and the cost effectiveness and sustainability in the medium- to long-term [5]. With respect to cost-effectiveness, there are various comparative studies that favour one or the other strategy [38-42]. Spraying, with one or two fumigation cycles, has been shown to be cheaper in the majority of the trials, when compared with the distribution of mosquito nets [39,41]. As the number of fumigation cycles increases (one or two per annum, depending on the endemicity), the cost of the strategy increases in comparison to re-treating mosquito nets [42].

The sustainability of any control strategy is conditioned by the availability of skilled national human resources and by the degree of economic investment, whether from private or state resources. The presence of economic interests in an area of high endemicity (e.g. the oil industry on the island of Bioko) fosters the implementation of control programmes with a high investment level, mainly with private resources [38]. The disappearance of those economic interests or the occurrence of a variety of internal phenomena (war, changes in government, etc...) may result in the control activities being interrupted. In the case of IRS, it has been noted that an interruption of activity may lead to the appearance of serious epidemic outbreaks, as was the case of South Africa in 1996 [43].

## Authors' contributions

GP was involved in the design of the second survey, participated in the data collection and drafted the manuscript. MD was involved in the design of the surveys, performed the statistical analysis and interpretation, and drafted the manuscript. EC was involved in the design of the first survey, participated in the data collection and helped to draft the manuscript. LM was involved in the design of both surveys, participated in the collection of the data and helped to draft the manuscript. ML, CM, JO and AN participated in the collection of the data and carried out the laboratory diagnosis. JR participated in the design, the interpretation of statistical analysis and drafted the manuscript. AB participated in the design of the surveys and has given approval of the version to be published. JC participated in the design, the interpretation of statistical analysis, and coordinated the draft of the manuscript. All authors read and approved the final manuscript.
